# The Korean Bird Information System (KBIS) through open and public participation

**DOI:** 10.1186/1471-2105-10-S15-S11

**Published:** 2009-12-03

**Authors:** In-Hwan Paik, Jeongheui Lim, Byung-Sun Chun, Seon-Duck Jin, Jae-Pyoung Yu, Joon-Woo Lee, Jong Bhak, Woon-Kee Paek

**Affiliations:** 1 Division of Natural History, National Science Museum, Daejeon, 305-705, Korea; 2 Korean BioInformation Center, Korea Research Institute of Bioscience and Biotechnology, Daejeon, 305-806, Korea; 3 Department of Environment Forest Resources, College of Agriculture & Life Science, Chungnam National University, 79 Daehangno, Yuseong-gu, Daejeon 305-764, Korea

## Abstract

**Background:**

The importance of biodiversity conservation has been increasing steadily due to its benefits to human beings. Recently, producing and managing biodiversity databases have become much easier because of the information technology (IT) advancement. This made the general public's participation in biodiversity conservation much more practical than ever. For example, an openfree web service can be devised for a wider spectrum of people to collaborate with each other for sharing biodiversity information. Bird migration is one such area of the collaboration. Korean migratory birds are usually traceable in the important routes of the East Asian-Australia Flyway (EAAF), and they play a key role as an environmental change indicator of the Earth. Therefore, the preservation of migratory birds requires an information system which involves a broader range of voluntary and interactive knowledge network to process bird information production, circulation, and dissemination.

**Results:**

The Korean Bird Information System (KBIS) aims to construct a cooperative partnership domestically and internationally through the acquisition, management, and sharing of Korean bird information involving both expert and non-expert groups. KBIS has six goals: data standard, system linkage, data diversity, utilization, bird knowledge network, and statistics. The key features of KBIS are to provide a simple search, gallery (photographs), and community to lead the participation of numerous non-experts, especially amateur bird watchers. The function of real-time observation data submission through the internet has been accomplished. It also provides bird banding database, statistics, and taxon network for experts. Especially, the statistics part provides the user with easy understanding of ecological trends of species based on the time and region.

**Conclusion:**

KBIS is a tool for the conservation and management of bird diversity and ecosystem that encourages users to participate by providing the openfree data access and real-time data input web-interface. It will enhance bird knowledge networking activities locally, nationally, and internationally. In addition, it provides opportunities to enhance the public awareness for the preservation of bird diversity and species information in relevant localities through the database construction and networking activities. It can be found at http://korbird.naris.go.kr.

## Background

Korea is located in the midst of the eastern Asian and Australian migratory bird flight paths [1] which have been serving as an important habitat for over 50 million water birds, equivalent to about 250 populations. However, because of coastal land reclamation and development policies, the Korean wetlands have been rapidly decreasing [2]. As birds migrating across national boundaries are at the top echelon of the food chain, the preservation and management of bird species requires a global effort.

Birds serve an important role as an indicator of the earth's environmental issues [3-5], pollution [6,7], and habitat environment [8]. As indicator species, birds require cooperative monitoring network with not only expert groups but the general public [9] as well. Through such cooperation, the construction of a bird information network has been actively carrying out in the world.

As an exemplary case, the Avian Knowledge Network (AKN) [10] is an outgrowth of voluntary participation of citizens to build databases for bird species and habitat information, thereby contributing to effective and efficient bird preservation and management. Recently, the knowledge associated with pre-existing knowledge management [11] have shifted their strategy which focused on technicality and efficiency before and this shift in strategy further developed into a two-way feedback structure as a knowledge network in the form of self-organization [12] and co-evolution [13] of knowledge, which in fact borrowed its concept from the ecological principles. In this sense, the AKN is striving to construct a bird knowledge network, a voluntary knowledge community [14] by occasioning the huge observational information supplied by birth watchers to be shared with ornithologists, conservation biologists, and land managers through the utility and real-time accessibility rendered possible by eBird [15].

Currently, the development of a biodiversity information system which can be shared, accessible, and utilized by on-line is underway by various institutions globally [16]. This trend is attributable to the low costs incurred and the resulting high-efficiency for requisite research, collection, application, and publication of biodiversity information which in turn contributes to the preservation, sustainable use, and management of biodiversity resources [16,17].

Recently, web environments, such as Web 2.0 which is defining characteristics with users' participation and data sharing, have emphasized to maximize the utility and accessibility of the vast number of bird observations made by recreational and professional bird watchers. There are some contributing factors to promote such web environment: the development of GPS and digital cameras, public awareness concerning sustainable society, public science, Flicker [18], Youtube [19], and personal media. Especially, since biodiversity is involved with global partnerships, the aforementioned web environment is perfectly in accord with the necessity for the development of biodiversity information system.

Based on this background, as a device for Korean bird diversity preservation and management, we developed KBIS to access and share the bird sightings and locations with birding community made by experts and non-experts.

## Methods

### System development strategy

**Figure 1:**
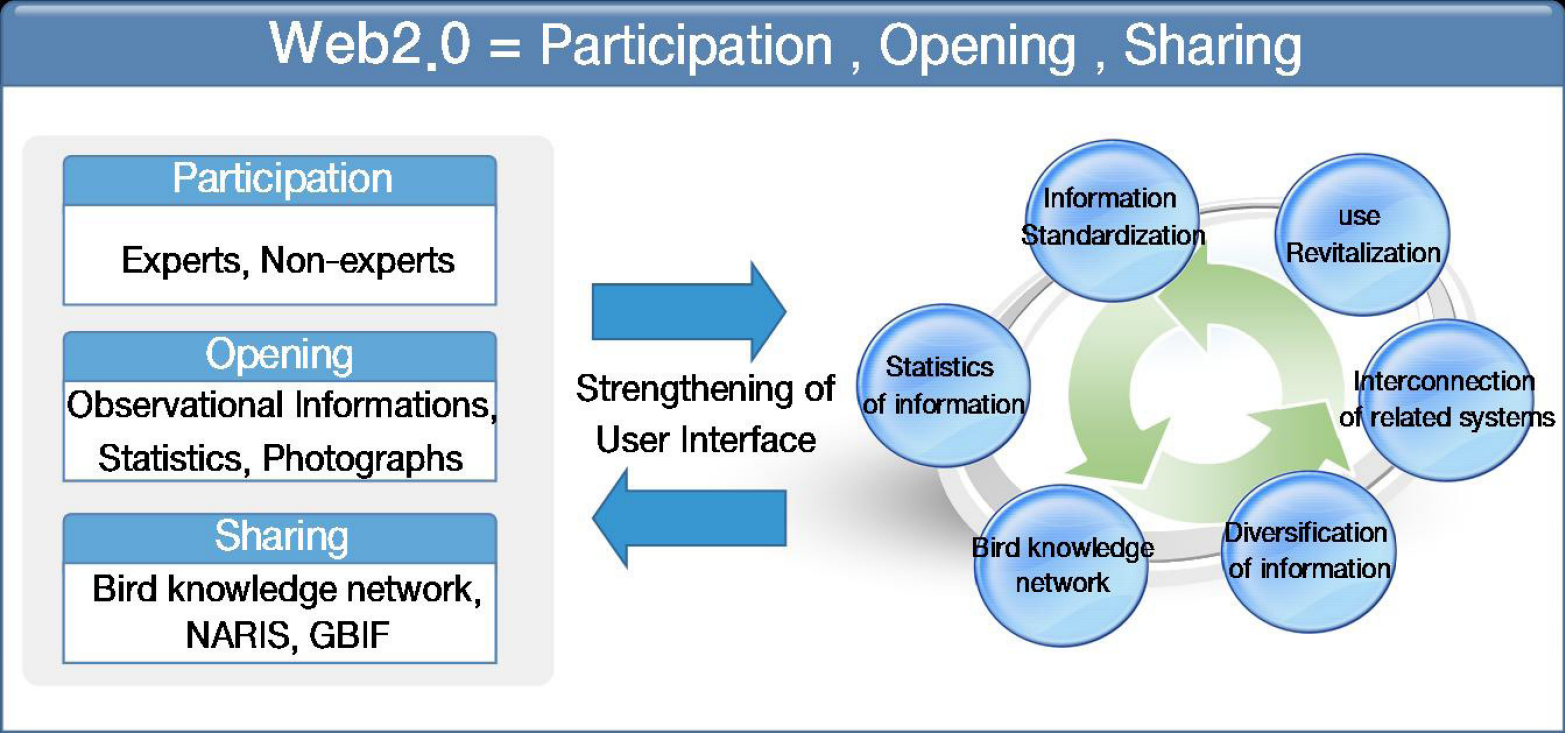
**The strategy of KBIS system development**.

KBIS was built on primarily observational information and photos supplied by voluntarily participating expert groups and non-experts interested in Korean birds, as well as birds that have migration routes through Korea. The intended users include not only expert groups but also non-expert groups who are interested in birds. Its purpose lies in establishing partnerships not limited to domestic groups but to international groups to share relevant data for preserving birds. As shown in Figure 1, KBIS has established six goals: data standardization of birds and habitats, linkage of related biodiversity information systems, diversity of data types, accelerating the collection and practical use of observational data by citizens, construction of bird knowledge networks emphasizing the issues of interesting populations and partnerships, and statistics of bird populations.

For the data standardization regarding bird species and habitats, the guidelines and classification systems from Global Biodiversity Information Facility (GBIF) [20] and Wetlands International have been adopted. The banding DB has been made compatible with those of domestic and international. To accelerate practical use encouraging the acquisition and application of observational data from the public, the functions of 'Gallery' and 'My page' have been adopted. The former is primarily comprised of photographs taken by domestic bird watchers. The latter is for exchanging opinions and comments to control any incorrect information and for setting up personal information. A statistics function has been developed to show the bird populations based on the time or geographical location and the most current location of each group and population.

### System architecture

**Figure 2:**
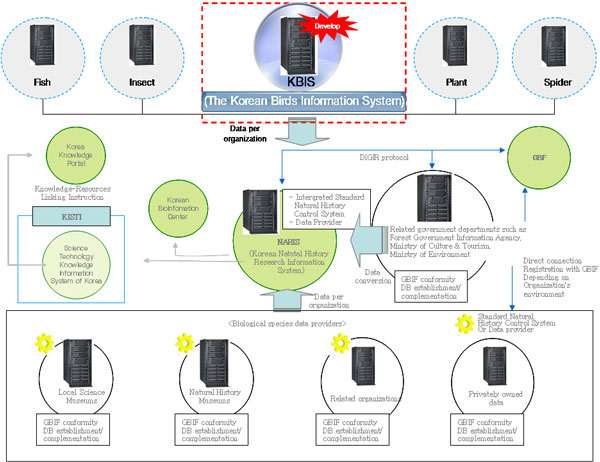
**KBIS system architecture**. All data sets configured by KBIS will be linked to NARIS, a Korean biodiversity information portal. All the NARIS data with that of KBIS data, through DiGIR protocol, will be linked to GBIF and other related information systems in Korea.

As shown in Figure 2, the bird information will be entered to database by conforming it to the data standard of the Korean Natural History Research Information System (NARIS) [21], a Korean biodiversity information portal system. KBIS used DiGIR protocol [22] recommended by GBIF, as a data exchange protocol, thereby sharing data and services with GBIF and NARIS.

### Data standard and protocol

**Figure 3:**
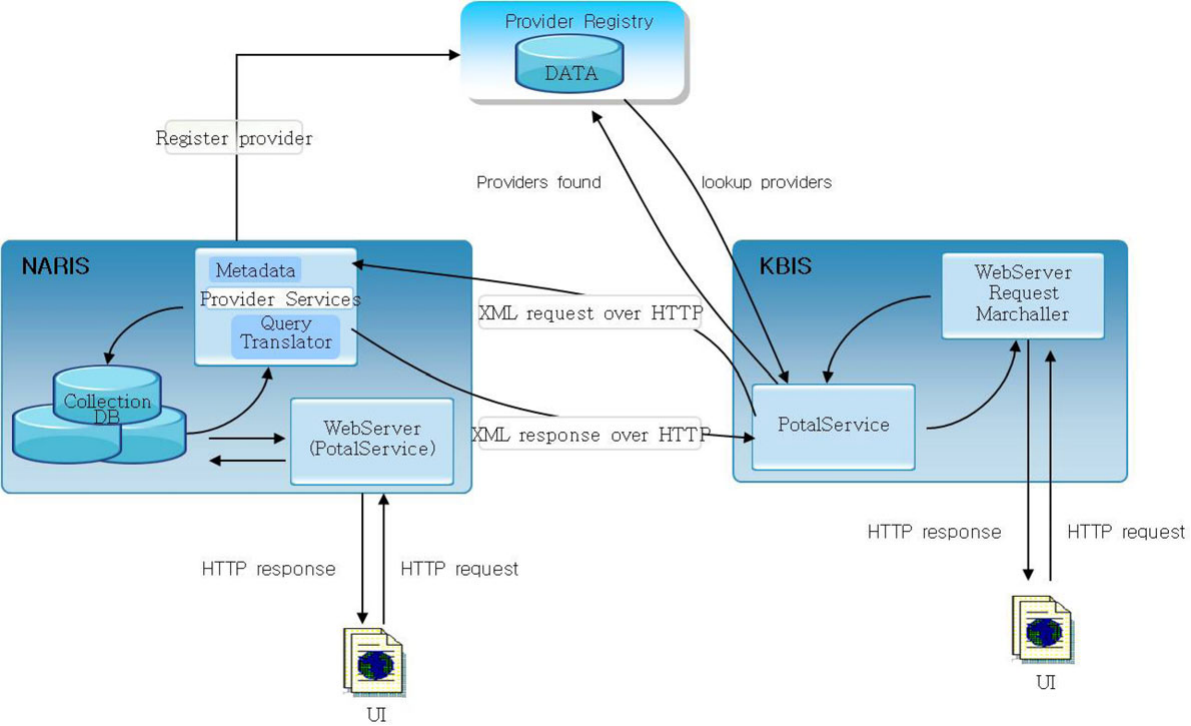
**Schematic diagram of the DiGIR protocol**. KBIS uses XML-based DiGIR protocol with HTTP as the transport mechanism and is designed to retrieve information from independently operating and de-synchronized databases. The list of bird species which is currently installed as a bird classification system based on NARIS, and the list has not yet been publicly recognized. Therefore, after the mapping of the two lists, the new one will be connected to the meta DB of NARIS.

The observational data in KBIS will be divided into species data and habitat data. For the species data, Darwin Core 2.0 [23] has been adopted as its data standard. The RIS Classification System for Wetland Type (The Information Sheet on Ramsar Wetlands) [24] provided by Wetlands International [25] which supervises the list of wetlands of international importance has been adopted for the habitat data standard. In an effort to search, link up, and integrate with the meta-database of its associated institutions, KBIS uses DiGIR protocol which is currently being used in international biodiversity community such as GBIF (Figure 3).

## Results and discussion

### User interface

The user interface of KBIS is mainly comprised of data search (e.g., integrated search and simple search in My Guide Book), search results, data input (in banding DB, gallery, and my page), input results, and statistics.

**Figure 4:**
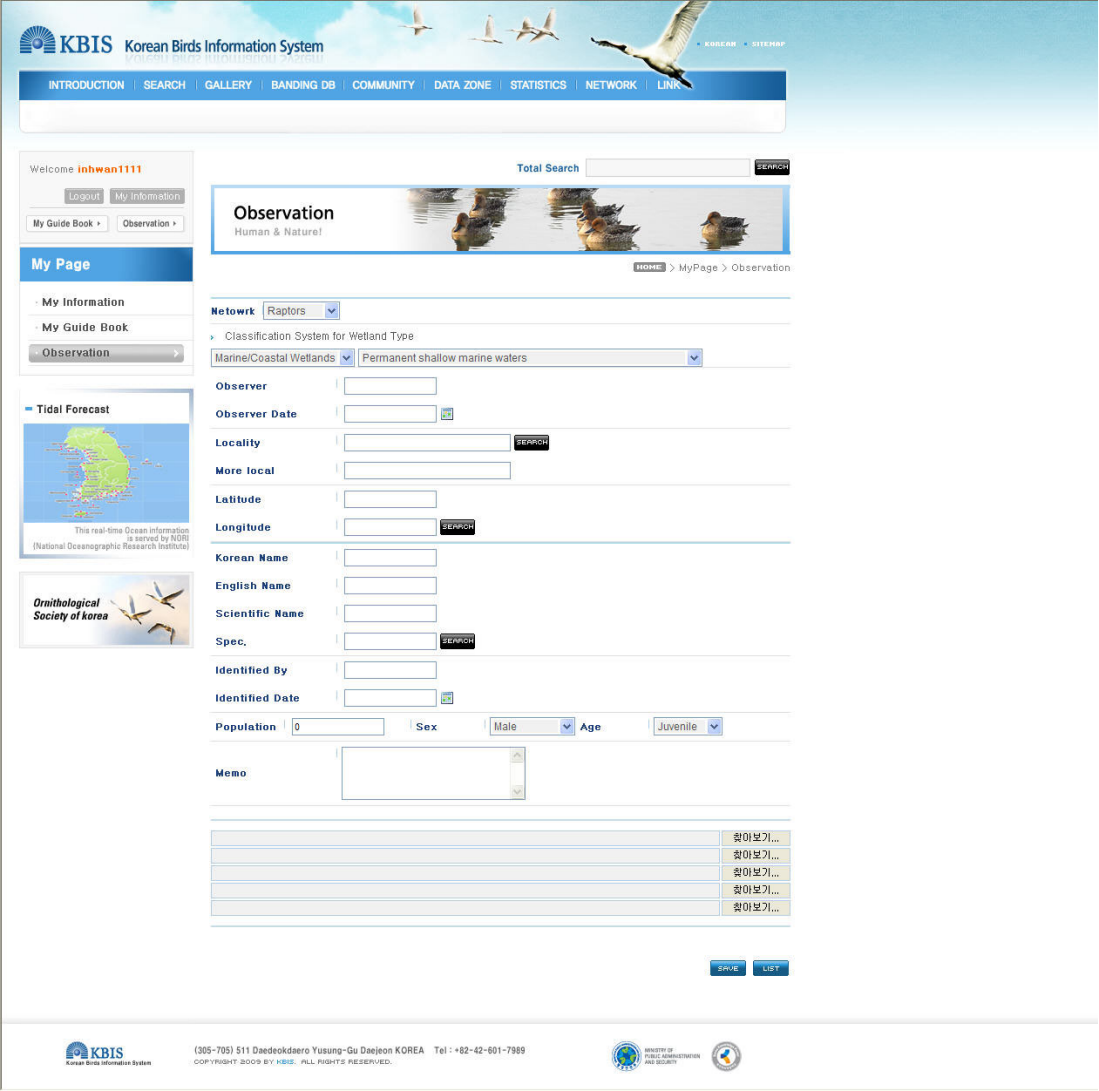
**An exemplary input screen for observational information**. The data input fields were constructed based on the data standard of Darwin Core2.0 and the classification system for wetland type of RIS (Ramsar Sites Information Service).

Search function can conduct an integrated search through simple input keywords for user convenience. The detailed search results are presented by species description, Google Map (API), gallery, banding data, and observational data. KBIS also has the following basic items to be entered: observer and species, location, administrative district, population number, gender, and photographs. Furthermore, a separate item relating to wetlands classification for the purposes of gathering information on waterbird habitats which cannot work without international partnerships is also included. This enables statistical analysis for the species habitation tendencies, based on the region and time (yearly or monthly) of population number and the type of wetlands (Figure 4).

### Key features

**Figure 5:**
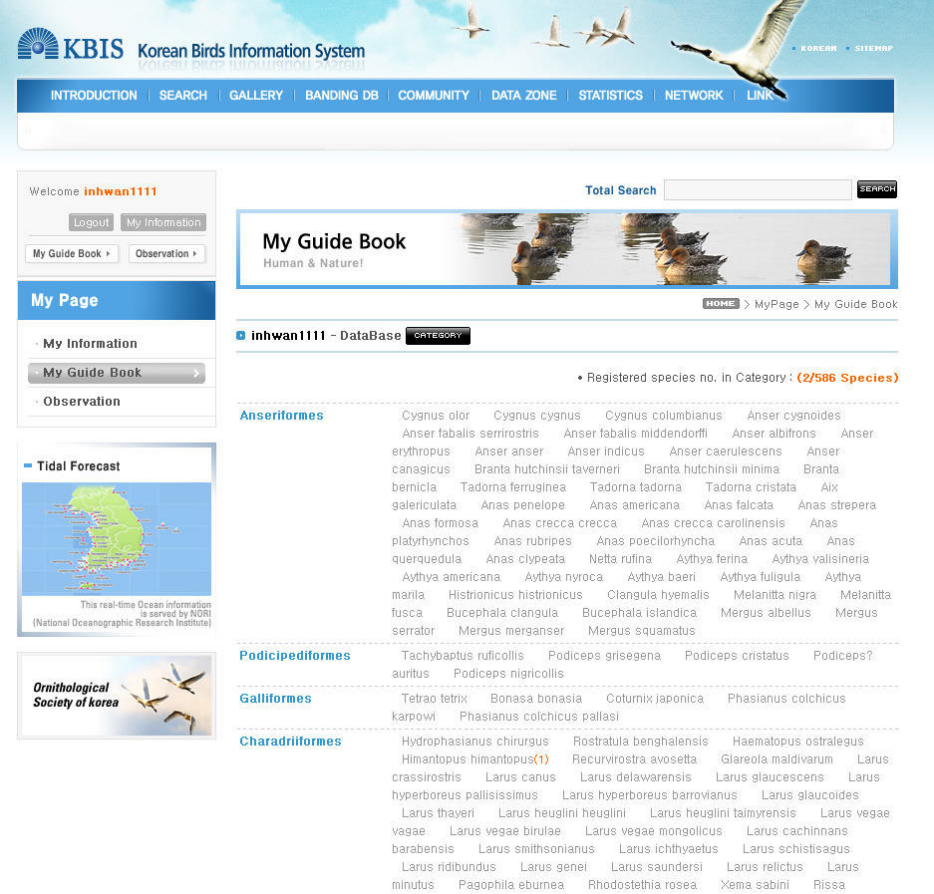
**Screenshot of 'My Guide Book' page**. The screen is showing the arranged list of bird classification based on the observations and photographs entered by a user.

By considering the internet usage patterns of Korean bird watchers, KBIS has emphasized the functions of 'Gallery' and 'My Guide Book'. In 'Gallery', various users' photographs being publicly accessible and questions regarding species description and photographing can be shared. In 'My Guide Book', the arranged list of bird classification based on the observations and photographs entered by a user is presented (Figure 5).

**Figure 6:**
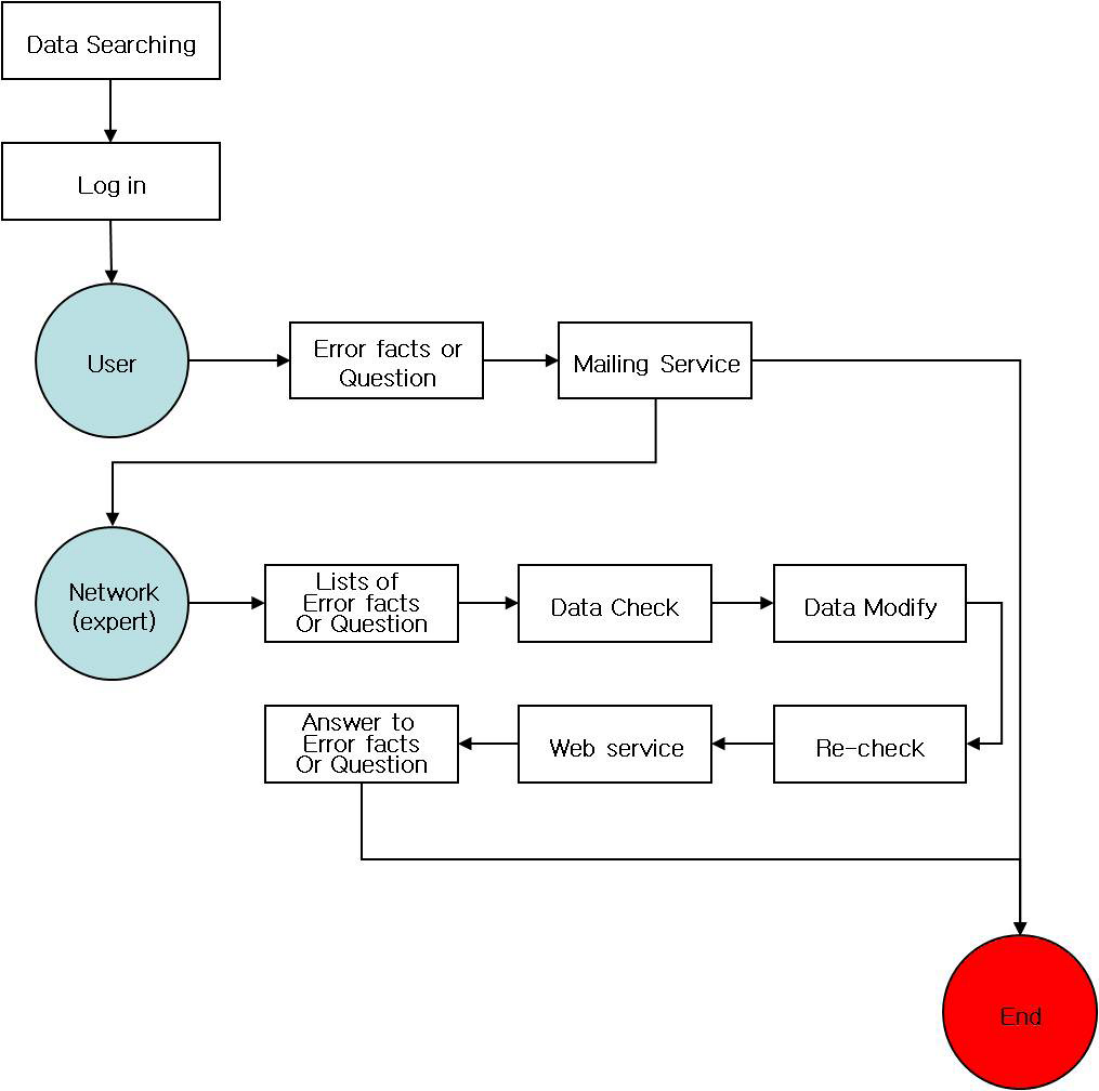
**Data quality management**. When there is any erroneous data input by users, they are corrected or revised using comments by other users and are also to be corrected or modified by experts through Q&A mailing services.

Since KBIS encourages the voluntary participation of recreational bird watchers, it is important to prevent incorrect data being integrated into the system. If there are any erroneous data from users, especially when it involves an uncertainty about the species name or pictures posted into the gallery, these data are to be corrected or revised using comments by user's review and are also to be corrected or modified by experts through Q&A mailing services (Figure 6).

**Figure 7:**
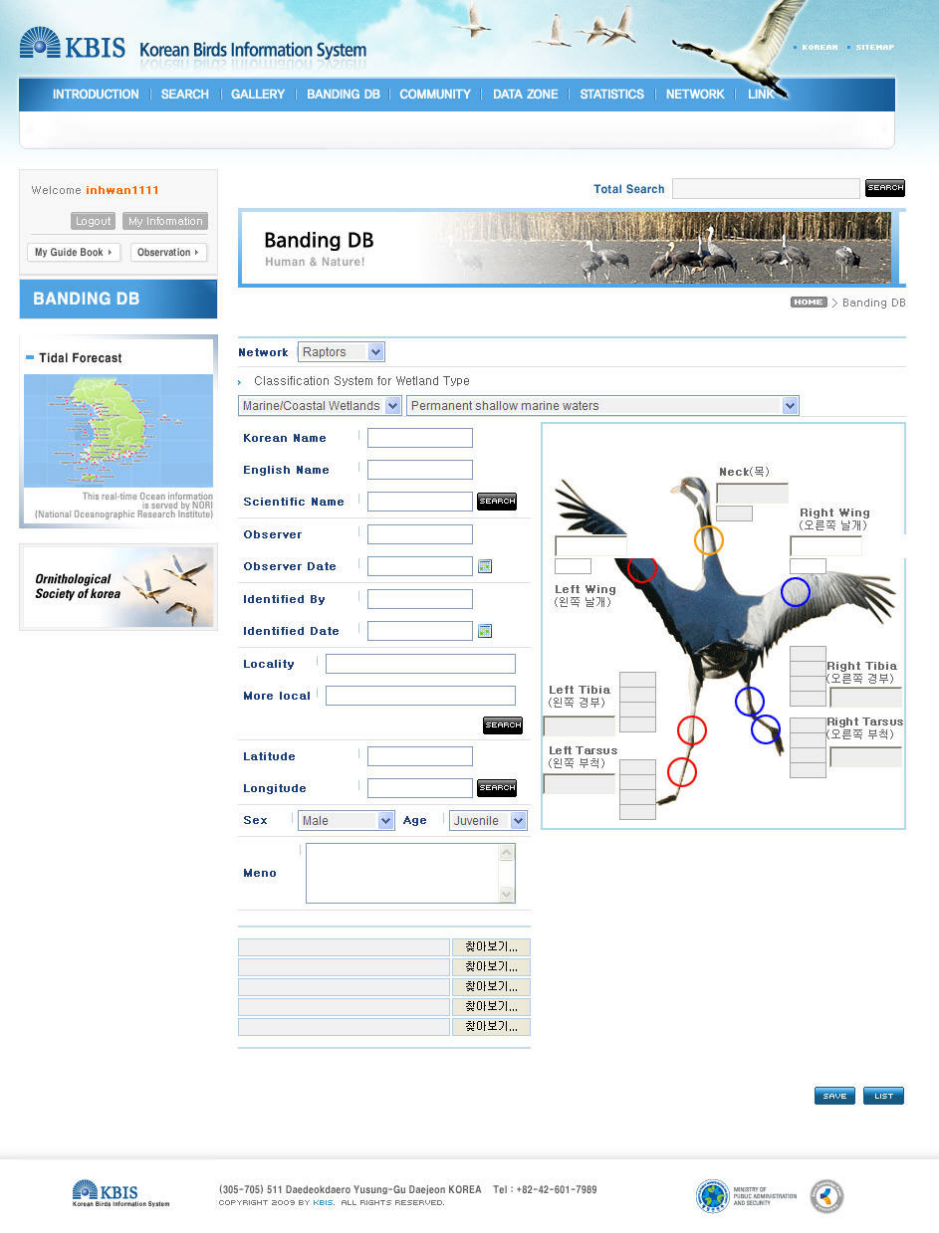
**An exemplary input screen of banding database**. The entry of banding DB consists of three sections (i.e., neck, wings, and legs - both left and right legs). The leg section consists of a combination of colors according to country and region and will be given a maximum of four boxes.

The banding DB input page has observational data entries, along with standard items of the bird banding type (Figure 7). In the entries, a selection box configured by taxon is provided, thereby inactivating the fill-in entry boxes to prevent any confusion over the different banding locations according to the different taxon groups (e.g., Anatidae, Cranes, Raptors and Shorebirds).

**Figure 8:**
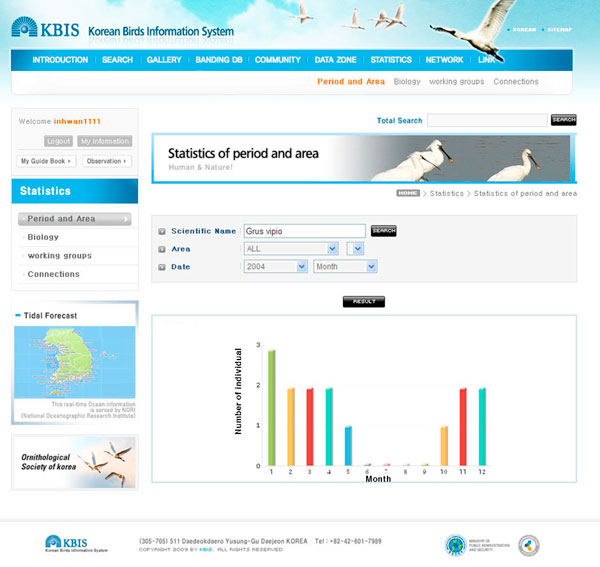
**An exemplary result screen of an statistical analysis**. The counting of searches by a user can show the size of the population on a monthly basis. The statistical function is accomplished to identify the variation and ecological tendencies of birds.

The statistical function can show bird population and colony changes based on the region and time, in keeping with user's intent. In addition, any information from a participant's regular monitoring on important habitats, such as bird fauna of a region and some threatening factors on birds' habitats, will be actively and widely referenced and utilized for strengthening international collaboration for bird protection, educational programs for enhancing public awareness, and promotional activities (Figure 8).

### Prospects

The future development of KBIS will include several new functions. First, the bird species identification program by its morphology will be developed to encourage easier participation of inexperienced users. Second, efforts will be made to increase the collection of various contents through the linkage with NARIS and GBIF. Third, KBIS will enforce the data error management function. Because the generation, publication, and utilization of the extensive bird knowledge network with many participation by the general public will be more required for data reliability. Finally, we plan to implement the mobile application interface so that researchers can easily and rapidly deposit and monitor data in real time for sampling locations, collections, and observations.

## Conclusion

KBIS's goal is to maximize the utility and accessibility of the vast number of bird observations, based on the interaction between recreational and professional bird watchers. KBIS has adopted international guidelines, such as Darwin Core 2.0 and DiGIR protocol, for data standard and exchange protocol. For the data quality control, the entered data are accompanied by comments and go through the verification by expert's reviews. The ecological information of bird was generated from observational and habitat data. The statistical function based on the region and time is accomplished to identify the variation and ecological tendencies of birds. With these various functions, KBIS are expected to play a great role in the conservation and protection of birds, collaborative partnerships, and environmental governance to the biodiversity community.

## Competing interests

The authors declare that they have no competing interests.

## Authors' contributions

WKP directed the study and helped draft the manuscript. BSC, SDJ, JB, JWL and JPY were involved in reviewing and critically revising it for intellectual content. IHP and JHL conceived the study and wrote the manuscript. All authors read and approved the final manuscript.

## Note

Other papers from the meeting have been published as part of *BMC Genomics* Volume 10 Supplement 3, 2009: Eighth International Conference on Bioinformatics (InCoB2009): Computational Biology, available online at http://www.biomedcentral.com/1471-2164/10?issue=S3.
